# 4-(1,3-Benzodioxol-5-yl)-2-oxo-1,2,5,6-tetra­hydro­benzo[*h*]quinoline-3-carbo­nitrile

**DOI:** 10.1107/S1600536811033903

**Published:** 2011-08-27

**Authors:** Abdullah M. Asiri, Hassan M. Faidallah, Abdulrahman O. Al-Youbi, Khalid A. Alamry, Seik Weng Ng

**Affiliations:** a Chemistry Department, Faculty of Science, King Abdulaziz University, PO Box 80203 Jeddah, Saudi Arabia; b Department of Chemistry, University of Malaya, 50603 Kuala Lumpur, Malaysia

## Abstract

In the mol­ecule of the title compound, C_21_H_14_N_2_O_3_, the tetra­hydro­benzo[*h*]quinoline fused-ring system is buckled owing to the ethyl­ene –CH_2_CH_2_– fragment, the benzene ring and the pyridine ring being twisted by 24.3 (1)°. The ring of the benzodioxol system is bent away from the pyridine ring by 61.4 (1)° in order to avoid crowding the cyanide substituent. Two mol­ecules are linked by a pair of N—H⋯O hydrogen bonds to form a centrosymmetric dimer.

## Related literature

For background to the anti­cancer properties of this class of compounds, see: Rostom *et al.* (2011 ▶).
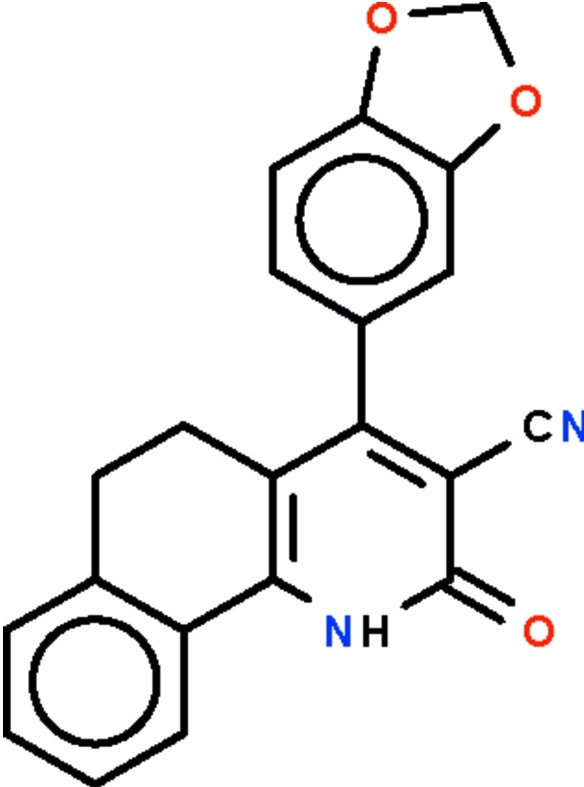


## Experimental

### 

#### Crystal data

C_21_H_14_N_2_O_3_
*M* *_r_* = 342.34
Monoclinic, 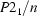

*a* = 7.6586 (3) Å
*b* = 16.5858 (5) Å
*c* = 13.3220 (6) Å
β = 104.164 (4)°
*V* = 1640.77 (11) Å^3^
*Z* = 4
Cu *K*α radiation
μ = 0.77 mm^−1^
*T* = 100 K
0.30 × 0.20 × 0.05 mm

#### Data collection

Agilent SuperNova Dual diffractometer with Atlas detector
Absorption correction: multi-scan (*CrysAlis PRO*; Agilent, 2010 ▶) *T*_min_ = 0.802, *T*_max_ = 0.963
6078 measured reflections
3241 independent reflections
2962 reflections with *I* > 2σ(*I*)
*R*_int_ = 0.014

#### Refinement

*R*[*F* ^2^ > 2σ(*F* ^2^)] = 0.036
*wR*(*F* ^2^) = 0.100
*S* = 1.02
3241 reflections
239 parameters
H atoms treated by a mixture of independent and constrained refinement
Δρ_max_ = 0.21 e Å^−3^
Δρ_min_ = −0.22 e Å^−3^

### 

Data collection: *CrysAlis PRO* (Agilent, 2010 ▶); cell refinement: *CrysAlis PRO*; data reduction: *CrysAlis PRO*; program(s) used to solve structure: *SHELXS97* (Sheldrick, 2008 ▶); program(s) used to refine structure: *SHELXL97* (Sheldrick, 2008 ▶); molecular graphics: *X-SEED* (Barbour, 2001 ▶); software used to prepare material for publication: *publCIF* (Westrip, 2010 ▶).

## Supplementary Material

Crystal structure: contains datablock(s) global, I. DOI: 10.1107/S1600536811033903/xu5293sup1.cif

Structure factors: contains datablock(s) I. DOI: 10.1107/S1600536811033903/xu5293Isup2.hkl

Supplementary material file. DOI: 10.1107/S1600536811033903/xu5293Isup3.cml

Additional supplementary materials:  crystallographic information; 3D view; checkCIF report

## Figures and Tables

**Table 1:** Hydrogen-bond geometry (Å, °)

*D*—H⋯*A*	*D*—H	H⋯*A*	*D*⋯*A*	*D*—H⋯*A*
N1—H1⋯O1^i^	0.93 (2)	1.85 (2)	2.778 (1)	175 (2)

Symmetry code: (i) 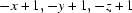.

**Fig. 1.**
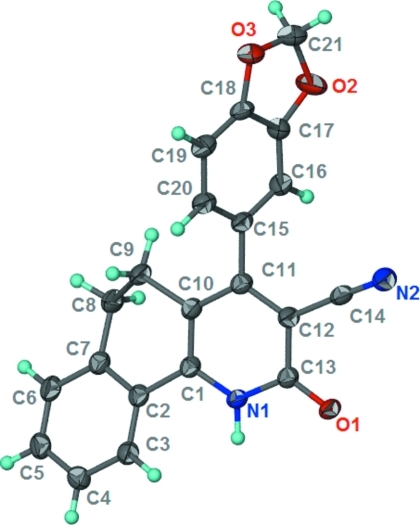
Thermal ellipsoid plot (Barbour, 2001) of C21H14N2O3 at the 70% probability level; hydrogen atoms are drawn as spheres of arbitrary radius.

## supplementary crystallographic information

### Comment

The compound (Scheme I) belongs to a series of cyano-pyridinones that have been evaluated for their anticancer properties (Rostom *et al.*, 2011). The tetrahydrobenzo[*h*]quinoline fused-ring system is buckled owing to the ethylene –CH_2_CH_2_– fragment, the benzene ring and the pyridine ring being twisted by 24.3 (1)°. The 4-subsituted aromatic ring is bent away from the pyridine ring by 61.4 (1) ° in order to avoid crowding the cyanide substituent (Fig. 1). Two molecules are linked by an N—H···O hydrogen bonds to form a centrosymmetric dimer (Table 1).

### Experimental

A mixture of piperonaldehyde (1.50 g, 10 mmol), 1-tetralone (1.46 g, 10 mmol), ethyl cyanoacetate (1.1 g, 10 mmol) and ammonium acetate (6.2 g, 80 mmol) in absolute ethanol (50 ml) was refluxed for 6 h. The reaction mixture was allowed to cool, and the yellow precipitate that formed was filtered, washed with water, dried and recrystallized from ethanol; m.p. 593–595 K.

### Refinement

Carbon- and nitrogen-bound H atoms were placed in calculated positions [C—H 0.95 to 0.99 Å, *U*_iso_(H) = 1.2*U*_eq_(C)] and were included in the refinement in the riding model approximation.

The amino H atom was located in a difference Fourier map and was freely refined.

### Crystal data

**Table:**

C_21_H_14_N_2_O_3_	*F*(000) = 712
*M**_r_* = 342.34	*D*_x_ = 1.386 Mg m^−^^3^
Monoclinic, *P*2_1_/*n*	Cu *K*α radiation, λ = 1.54184 Å
Hall symbol: -P 2yn	Cell parameters from 3826 reflections
*a* = 7.6586 (3) Å	θ = 3.4–74.1°
*b* = 16.5858 (5) Å	µ = 0.77 mm^−^^1^
*c* = 13.3220 (6) Å	*T* = 100 K
β = 104.164 (4)°	Prism, yellow
*V* = 1640.77 (11) Å^3^	0.30 × 0.20 × 0.05 mm
*Z* = 4	

### Data collection

**Table:**

Agilent SuperNova Dual diffractometer with Atlas detector	3241 independent reflections
Radiation source: SuperNova (Cu) X-ray Source	2962 reflections with *I* > 2σ(*I*)
mirror	*R*_int_ = 0.014
Detector resolution: 10.4041 pixels mm^-1^	θ_max_ = 74.3°, θ_min_ = 4.3°
ω scans	*h* = −9→9
Absorption correction: multi-scan (*CrysAlis PRO*; Agilent, 2010)	*k* = −20→18
*T*_min_ = 0.802, *T*_max_ = 0.963	*l* = −12→16
6078 measured reflections	

### Refinement

**Table:**

Refinement on *F*^2^	Primary atom site location: structure-invariant direct methods
Least-squares matrix: full	Secondary atom site location: difference Fourier map
*R*[*F*^2^ > 2σ(*F*^2^)] = 0.036	Hydrogen site location: inferred from neighbouring sites
*wR*(*F*^2^) = 0.100	H atoms treated by a mixture of independent and constrained refinement
*S* = 1.02	*w* = 1/[σ^2^(*F*_o_^2^) + (0.0584*P*)^2^ + 0.3916*P*] where *P* = (*F*_o_^2^ + 2*F*_c_^2^)/3
3241 reflections	(Δ/σ)_max_ = 0.001
239 parameters	Δρ_max_ = 0.21 e Å^−^^3^
0 restraints	Δρ_min_ = −0.22 e Å^−^^3^

### Fractional atomic coordinates and isotropic or equivalent isotropic displacement parameters (Å^2^)

**Table:**

	*x*	*y*	*z*	*U*_iso_*/*U*_eq_	
O1	0.70432 (10)	0.53335 (5)	0.56250 (6)	0.0240 (2)	
O2	1.17132 (13)	0.94538 (6)	0.60416 (10)	0.0444 (3)	
O3	1.07114 (12)	1.02058 (5)	0.72509 (8)	0.0325 (2)	
N1	0.46189 (12)	0.61383 (6)	0.49726 (7)	0.0200 (2)	
H1	0.400 (2)	0.5663 (10)	0.4755 (12)	0.036 (4)*	
N2	1.07002 (14)	0.65042 (6)	0.67925 (9)	0.0305 (3)	
C1	0.38331 (15)	0.68751 (7)	0.47365 (8)	0.0197 (2)	
C2	0.19123 (15)	0.69051 (7)	0.41849 (9)	0.0210 (2)	
C3	0.07235 (15)	0.62711 (7)	0.42051 (9)	0.0233 (2)	
H3	0.1138	0.5799	0.4594	0.028*	
C4	−0.10680 (16)	0.63304 (7)	0.36557 (10)	0.0270 (3)	
H4	−0.1877	0.5899	0.3671	0.032*	
C5	−0.16769 (16)	0.70217 (8)	0.30833 (10)	0.0288 (3)	
H5	−0.2899	0.7060	0.2705	0.035*	
C6	−0.05002 (16)	0.76546 (7)	0.30652 (9)	0.0268 (3)	
H6	−0.0922	0.8124	0.2672	0.032*	
C7	0.12963 (16)	0.76085 (7)	0.36181 (9)	0.0232 (2)	
C8	0.26177 (16)	0.82766 (7)	0.36083 (10)	0.0282 (3)	
H8A	0.1958	0.8789	0.3413	0.034*	
H8B	0.3302	0.8158	0.3084	0.034*	
C9	0.39269 (16)	0.83692 (7)	0.46727 (10)	0.0271 (3)	
H9A	0.4852	0.8778	0.4636	0.032*	
H9B	0.3266	0.8555	0.5182	0.032*	
C10	0.48223 (15)	0.75711 (7)	0.50141 (9)	0.0210 (2)	
C11	0.66347 (15)	0.75062 (7)	0.55957 (9)	0.0212 (2)	
C12	0.74037 (15)	0.67503 (7)	0.57985 (9)	0.0212 (2)	
C13	0.63948 (15)	0.60256 (7)	0.54712 (8)	0.0201 (2)	
C14	0.92402 (15)	0.66289 (7)	0.63508 (9)	0.0228 (3)	
C15	0.76691 (15)	0.82441 (6)	0.60147 (9)	0.0213 (2)	
C16	0.92690 (16)	0.84474 (7)	0.57344 (10)	0.0252 (3)	
H16	0.9722	0.8130	0.5261	0.030*	
C17	1.01415 (15)	0.91297 (7)	0.61812 (10)	0.0262 (3)	
C18	0.95379 (16)	0.95848 (7)	0.68946 (9)	0.0238 (2)	
C19	0.79788 (16)	0.94009 (7)	0.71780 (9)	0.0250 (3)	
H19	0.7560	0.9718	0.7665	0.030*	
C20	0.70363 (16)	0.87202 (7)	0.67114 (9)	0.0239 (2)	
H20	0.5935	0.8579	0.6875	0.029*	
C21	1.20431 (18)	1.01643 (8)	0.66633 (11)	0.0346 (3)	
H21A	1.1967	1.0647	0.6218	0.041*	
H21B	1.3263	1.0143	0.7134	0.041*	

### Atomic displacement parameters (Å^2^)

**Table:**

	*U*^11^	*U*^22^	*U*^33^	*U*^12^	*U*^13^	*U*^23^
O1	0.0226 (4)	0.0149 (4)	0.0296 (4)	0.0014 (3)	−0.0028 (3)	0.0014 (3)
O2	0.0336 (5)	0.0346 (5)	0.0716 (8)	−0.0158 (4)	0.0256 (5)	−0.0236 (5)
O3	0.0289 (4)	0.0224 (4)	0.0442 (5)	−0.0065 (3)	0.0051 (4)	−0.0121 (4)
N1	0.0196 (5)	0.0147 (4)	0.0226 (5)	−0.0005 (4)	−0.0007 (4)	−0.0004 (4)
N2	0.0250 (5)	0.0230 (5)	0.0375 (6)	0.0003 (4)	−0.0040 (4)	−0.0018 (4)
C1	0.0213 (5)	0.0186 (5)	0.0181 (5)	0.0018 (4)	0.0027 (4)	−0.0002 (4)
C2	0.0204 (5)	0.0203 (5)	0.0205 (5)	0.0026 (4)	0.0018 (4)	−0.0029 (4)
C3	0.0224 (5)	0.0219 (6)	0.0240 (6)	0.0024 (4)	0.0027 (4)	−0.0020 (4)
C4	0.0216 (5)	0.0269 (6)	0.0306 (6)	−0.0011 (5)	0.0028 (5)	−0.0049 (5)
C5	0.0209 (5)	0.0296 (6)	0.0316 (6)	0.0046 (5)	−0.0019 (5)	−0.0058 (5)
C6	0.0250 (6)	0.0229 (6)	0.0281 (6)	0.0067 (5)	−0.0022 (5)	−0.0018 (5)
C7	0.0234 (5)	0.0209 (5)	0.0235 (5)	0.0030 (4)	0.0021 (4)	−0.0032 (4)
C8	0.0254 (6)	0.0209 (6)	0.0338 (7)	0.0033 (4)	−0.0013 (5)	0.0056 (5)
C9	0.0245 (6)	0.0164 (5)	0.0362 (7)	0.0022 (4)	−0.0005 (5)	−0.0007 (5)
C10	0.0218 (5)	0.0174 (5)	0.0226 (5)	0.0020 (4)	0.0031 (4)	−0.0010 (4)
C11	0.0233 (5)	0.0181 (5)	0.0211 (5)	0.0002 (4)	0.0031 (4)	−0.0015 (4)
C12	0.0200 (5)	0.0189 (5)	0.0219 (5)	−0.0004 (4)	−0.0003 (4)	0.0000 (4)
C13	0.0210 (5)	0.0181 (5)	0.0190 (5)	0.0012 (4)	0.0007 (4)	0.0010 (4)
C14	0.0250 (6)	0.0150 (5)	0.0260 (6)	−0.0006 (4)	0.0016 (5)	−0.0020 (4)
C15	0.0226 (5)	0.0157 (5)	0.0222 (5)	0.0007 (4)	−0.0010 (4)	−0.0002 (4)
C16	0.0243 (5)	0.0214 (6)	0.0291 (6)	0.0003 (4)	0.0050 (5)	−0.0060 (5)
C17	0.0219 (5)	0.0217 (6)	0.0343 (6)	−0.0022 (4)	0.0054 (5)	−0.0026 (5)
C18	0.0267 (6)	0.0147 (5)	0.0258 (6)	−0.0017 (4)	−0.0015 (4)	−0.0020 (4)
C19	0.0315 (6)	0.0195 (5)	0.0232 (6)	0.0009 (4)	0.0051 (5)	−0.0026 (4)
C20	0.0266 (6)	0.0189 (5)	0.0255 (6)	−0.0012 (4)	0.0050 (5)	0.0000 (4)
C21	0.0342 (7)	0.0237 (6)	0.0449 (8)	−0.0085 (5)	0.0081 (6)	−0.0066 (6)

### Geometric parameters (Å, °)

**Table:**

O1—C13	1.2477 (14)	C8—C9	1.5309 (17)
O2—C17	1.3718 (15)	C8—H8A	0.9900
O2—C21	1.4269 (16)	C8—H8B	0.9900
O3—C18	1.3730 (14)	C9—C10	1.5088 (15)
O3—C21	1.4312 (17)	C9—H9A	0.9900
N1—C1	1.3645 (14)	C9—H9B	0.9900
N1—C13	1.3725 (14)	C10—C11	1.4185 (15)
N1—H1	0.931 (17)	C11—C12	1.3834 (15)
N2—C14	1.1477 (15)	C11—C15	1.4902 (15)
C1—C10	1.3807 (15)	C12—C14	1.4331 (15)
C1—C2	1.4756 (15)	C12—C13	1.4377 (15)
C2—C3	1.3955 (16)	C15—C20	1.3928 (16)
C2—C7	1.4068 (16)	C15—C16	1.4065 (17)
C3—C4	1.3916 (16)	C16—C17	1.3736 (16)
C3—H3	0.9500	C16—H16	0.9500
C4—C5	1.3928 (18)	C17—C18	1.3790 (17)
C4—H4	0.9500	C18—C19	1.3718 (17)
C5—C6	1.3875 (18)	C19—C20	1.4010 (16)
C5—H5	0.9500	C19—H19	0.9500
C6—C7	1.3948 (15)	C20—H20	0.9500
C6—H6	0.9500	C21—H21A	0.9900
C7—C8	1.5029 (17)	C21—H21B	0.9900
			
C17—O2—C21	106.35 (10)	C1—C10—C11	118.83 (10)
C18—O3—C21	105.58 (9)	C1—C10—C9	118.27 (10)
C1—N1—C13	124.23 (9)	C11—C10—C9	122.89 (10)
C1—N1—H1	121.6 (10)	C12—C11—C10	119.24 (10)
C13—N1—H1	114.0 (10)	C12—C11—C15	120.61 (10)
N1—C1—C10	120.33 (10)	C10—C11—C15	120.11 (10)
N1—C1—C2	118.34 (10)	C11—C12—C14	123.03 (10)
C10—C1—C2	121.33 (10)	C11—C12—C13	121.79 (10)
C3—C2—C7	120.03 (10)	C14—C12—C13	115.17 (10)
C3—C2—C1	122.72 (10)	O1—C13—N1	120.82 (10)
C7—C2—C1	117.25 (10)	O1—C13—C12	123.82 (10)
C4—C3—C2	120.04 (11)	N1—C13—C12	115.36 (9)
C4—C3—H3	120.0	N2—C14—C12	177.69 (12)
C2—C3—H3	120.0	C20—C15—C16	120.58 (10)
C5—C4—C3	120.07 (11)	C20—C15—C11	118.39 (10)
C5—C4—H4	120.0	C16—C15—C11	121.02 (10)
C3—C4—H4	120.0	C17—C16—C15	116.46 (11)
C6—C5—C4	120.03 (11)	C17—C16—H16	121.8
C6—C5—H5	120.0	C15—C16—H16	121.8
C4—C5—H5	120.0	O2—C17—C16	127.88 (11)
C5—C6—C7	120.68 (11)	O2—C17—C18	109.40 (10)
C5—C6—H6	119.7	C16—C17—C18	122.68 (11)
C7—C6—H6	119.7	O3—C18—C19	127.72 (11)
C6—C7—C2	119.13 (11)	O3—C18—C17	110.33 (11)
C6—C7—C8	122.30 (10)	C19—C18—C17	121.95 (11)
C2—C7—C8	118.55 (10)	C18—C19—C20	116.47 (11)
C7—C8—C9	110.84 (10)	C18—C19—H19	121.8
C7—C8—H8A	109.5	C20—C19—H19	121.8
C9—C8—H8A	109.5	C15—C20—C19	121.81 (11)
C7—C8—H8B	109.5	C15—C20—H20	119.1
C9—C8—H8B	109.5	C19—C20—H20	119.1
H8A—C8—H8B	108.1	O2—C21—O3	107.97 (10)
C10—C9—C8	109.72 (10)	O2—C21—H21A	110.1
C10—C9—H9A	109.7	O3—C21—H21A	110.1
C8—C9—H9A	109.7	O2—C21—H21B	110.1
C10—C9—H9B	109.7	O3—C21—H21B	110.1
C8—C9—H9B	109.7	H21A—C21—H21B	108.4
H9A—C9—H9B	108.2		
			
C13—N1—C1—C10	−1.05 (17)	C10—C11—C12—C13	−2.52 (17)
C13—N1—C1—C2	179.18 (10)	C15—C11—C12—C13	175.21 (10)
N1—C1—C2—C3	22.74 (16)	C1—N1—C13—O1	−177.03 (10)
C10—C1—C2—C3	−157.03 (11)	C1—N1—C13—C12	3.52 (16)
N1—C1—C2—C7	−156.96 (10)	C11—C12—C13—O1	178.92 (11)
C10—C1—C2—C7	23.27 (16)	C14—C12—C13—O1	−1.53 (17)
C7—C2—C3—C4	0.69 (17)	C11—C12—C13—N1	−1.66 (16)
C1—C2—C3—C4	−179.00 (10)	C14—C12—C13—N1	177.90 (10)
C2—C3—C4—C5	0.11 (18)	C12—C11—C15—C20	−116.89 (13)
C3—C4—C5—C6	−0.38 (19)	C10—C11—C15—C20	60.82 (15)
C4—C5—C6—C7	−0.15 (19)	C12—C11—C15—C16	61.65 (16)
C5—C6—C7—C2	0.94 (17)	C10—C11—C15—C16	−120.64 (12)
C5—C6—C7—C8	179.27 (12)	C20—C15—C16—C17	−0.08 (17)
C3—C2—C7—C6	−1.21 (17)	C11—C15—C16—C17	−178.59 (11)
C1—C2—C7—C6	178.50 (10)	C21—O2—C17—C16	179.29 (13)
C3—C2—C7—C8	−179.60 (11)	C21—O2—C17—C18	−2.82 (15)
C1—C2—C7—C8	0.11 (16)	C15—C16—C17—O2	179.76 (12)
C6—C7—C8—C9	143.06 (11)	C15—C16—C17—C18	2.13 (18)
C2—C7—C8—C9	−38.60 (15)	C21—O3—C18—C19	−176.17 (12)
C7—C8—C9—C10	54.40 (14)	C21—O3—C18—C17	4.47 (13)
N1—C1—C10—C11	−3.38 (17)	O2—C17—C18—O3	−1.08 (14)
C2—C1—C10—C11	176.38 (10)	C16—C17—C18—O3	176.94 (11)
N1—C1—C10—C9	175.95 (10)	O2—C17—C18—C19	179.52 (11)
C2—C1—C10—C9	−4.29 (16)	C16—C17—C18—C19	−2.46 (19)
C8—C9—C10—C1	−34.42 (15)	O3—C18—C19—C20	−178.70 (11)
C8—C9—C10—C11	144.89 (11)	C17—C18—C19—C20	0.59 (17)
C1—C10—C11—C12	5.05 (17)	C16—C15—C20—C19	−1.71 (17)
C9—C10—C11—C12	−174.25 (11)	C11—C15—C20—C19	176.83 (10)
C1—C10—C11—C15	−172.70 (10)	C18—C19—C20—C15	1.44 (17)
C9—C10—C11—C15	8.01 (17)	C17—O2—C21—O3	5.54 (15)
C10—C11—C12—C14	177.96 (10)	C18—O3—C21—O2	−6.13 (14)
C15—C11—C12—C14	−4.31 (18)		

### Hydrogen-bond geometry (Å, °)

**Table:**

*D*—H···*A*	*D*—H	H···*A*	*D*···*A*	*D*—H···*A*
N1—H1···O1^i^	0.93 (2)	1.85 (2)	2.778 (1)	175 (2)

Symmetry codes: (i) −*x*+1, −*y*+1, −*z*+1.
